# THE IMPACT OF SOCIAL DETERMINANTS OF HEALTH ON FRAILTY TRAJECTORIES OF OLDER ADULTS IN RURAL UNITED STATES

**DOI:** 10.1093/geroni/igae098.1710

**Published:** 2024-12-31

**Authors:** Hillary Spangler, Wenyi Xie, David Lynch, Feng-Chang Lin, John Batsis

**Affiliations:** University of North Carolina School of Medicine, Chapel Hill, North Carolina, United States; University of North Carolina, Chapel Hill, North Carolina, United States; University of North Carolina School of Medicine, Chapel Hill, North Carolina, United States; University of North Carolina, Chapel Hill, North Carolina, United States; University of North Carolina, Chapel Hill, North Carolina, United States

## Abstract

**Background:**

Frailty is a geriatric syndrome of physical decline that increases an older adult’s risk for vulnerability to physiological stress. We aim to determine the impact of social determinants of health (SDOH) on frailty trajectories of older adults in rural areas.

**Methods:**

We used data from the National Health and Aging Trend Study (2011-2020), a cohort of Medicare beneficiaries >65 years. Using cohorts from 2011 (n=8,245) and 2015 (n=8,334), individuals were categorized using Fried’s frailty phenotype. Rurality was defined using the Office of Management and Budget’s definition. Generalized estimation equations with generalized logit link function determined the impact of rurality and SDOH (transportation, family support, income, education) on frailty status.

**Results:**

Using the 2011 (n=8,245) and 2015 (n=8,334) cohorts, participants were 57.1% and 55.8% female, median age 70-74 years old, and 18.5% and 19.3% lived in rural areas. Being married, having a college education, and higher income were protective against frailty progression. Urban areas had lower odds developing frailty versus pre-frailty, yet hastened developing pre-frailty versus robust. Urban adults with transportation to doctor appointments had lower odds of frailty progression, though not significant (robust->frail (OR 0.80, SE 0.09, p=0.47) and robust->pre-frail (OR 0.83, SE 0.08, p=0.49)). Hispanic ethnicity was protective against progressive frailty robust->frail (OR 0.40, SE 0.14, p=0.008) or pre-frail->frail (OR 0.55, SE 0.13, p=0.01).

**Conclusion:**

Our findings suggest SDOH, race, and rurality may impact frailty trajectories. Future studies need to investigate the heterogeneity of aging in groups of older adults with similar SDOH and geography.

